# The need for treatment scale-up to impact HCV transmission in people who inject drugs in Montréal, Canada: a modelling study

**DOI:** 10.1186/s12879-017-2256-5

**Published:** 2017-02-21

**Authors:** Anthony Cousien, Pascale Leclerc, Carole Morissette, Julie Bruneau, Élise Roy, Viet Chi Tran, Yazdan Yazdanpanah, Joseph Cox

**Affiliations:** 1 IAME, UMR 1137, INSERM, F-75018 Paris, France; 2 IAME, UMR 1137, Univ Paris Diderot, Sorbonne Paris Cité, F-75018 Paris, France; 3 Direction régionale de santé publique du Centre intégré universitaire de santé et de services sociaux du Centre-Sud-de-l’Ile-de-Montréal, 1301 rue Sherbrooke est, Montréal, QC H2L 1M3 Canada; 4 Centre de recherche, Centre hospitalier de l’Université de Montréal (CRCHUM), 900 Saint-Denis, Montréal, QC H2X 0A9 Canada; 5 Faculté de médecine et des sciences de la santé, Université de Sherbrooke, Campus Longueuil, 150 place Charles-Le Moyne, Longueuil, QC J4K 0A8 Canada; 6 Laboratoire Paul Painlevé UMR CNRS 8524, UFR de Mathématiques, Université des Sciences et Technologies Lille 1, Cité Scientifique, Villeneuve d’Ascq, France; 7 Service des Maladies Infectieuses et Tropicales, Hôpital Bichat Claude Bernard, Paris, France; 8 Department of Epidemiology, Biostatistics and Occupational Health, McGill University, Purvis Hall, 1020 Pine Avenue West, Montreal, QC H3A 1A3 Canada; 9 Chronic Viral Illness Service, McGill University Health Centre, 1001 Decarie Blvd., Montreal, QC H4A3J1 Canada

**Keywords:** Dynamic model, HCV elimination, Treatment initiation criteria, Cascade of care, Direct-acting antiviral, People who inject drugs

## Abstract

**Background:**

HCV transmission remains high in people who inject drugs (PWID) in Montréal. New direct-acting antivirals (DAAs), highly effective and more tolerable than previous regimens, make a “Treatment as Prevention” (TasP) strategy more feasible. This study assesses how improvements in the cascade of care could impact hepatitis C burden among PWID in Montréal.

**Methods:**

We used a dynamic model to simulate HCV incidence and prevalence after 10 years, and cirrhosis complications after 10 and 40 years. Eight scenarios of improved cascade of care were examined.

**Results:**

Using a baseline incidence and prevalence of 22.1/100 person-years (PY) and 53.1%, implementing the current cascade of care using DAAs would lead to HCV incidence and prevalence estimates at 10 years of 9.4/100PY and 55.8%, respectively. Increasing the treatment initiation rate from 5%/year initially to 20%/year resulted in large decreases in incidence (6.4/100PY), prevalence (36.6%), and cirrhosis complications (−18%/-37% after 10/40 years). When restricting treatment to fibrosis level ≥ F2 instead of F0 (reference scenario), such decreases in HCV occurrence were unreachable. Improving the whole cascade of care led to the greatest effect by halving both the incidence and prevalence at 10 years, and the number of cirrhosis complications after 40 years.

**Conclusions:**

The current level of treatment access in Montréal is limiting a massive decrease in hepatitis C burden among PWID. A substantial treatment scale-up, regardless of fibrosis level, is necessary. While improving the rest of the cascade of care is necessary to optimize a TasP strategy and control the HCV epidemic, a treatment scale-up is first needed.

**Electronic supplementary material:**

The online version of this article (doi:10.1186/s12879-017-2256-5) contains supplementary material, which is available to authorized users.

## Background

Drug injection is the main transmission route for hepatitis C virus (HCV) in high income countries [1]. The number of active people who inject drugs (PWID) (defined as injecting in the past six months) in Montréal is estimated to be 4,000 [2]. According to regional surveillance data (SurvUDI network), approximately 70% of this population has been exposed (antibody positive) to HCV [3] and the number of new infections remains high: 22.1/100 persons-years (PY) for the 2010–2013 period (unpublished SurvUDI data).

Access to HCV treatment remains limited in this population. Several components of the HCV cascade of care may explain poor treatment uptake. During 2003–2011, 23% of the infected PWID reported they were not aware of their infection; among those who were aware, 45% reported a physician consultation in the past 6 months, and 12% initiated HCV treatment [3]. There may be reluctance on the part of physicians to initiate antiviral treatment in PWID [4]. Precarious living conditions and other co-morbidities (e.g., psychiatric disorders) may have been identified as barriers to treatment initiation [5]. Also, uncontrolled substance use often constitutes a treatment barrier and many physicians prefer to treat PWID who participate in opiate substitution programs [4]. Until recently, the standard antiviral treatment regimen for HCV (dual therapy pegylated interferon plus ribavirin) implied numerous challenges. The regimen required a treatment duration of 24 to 48 weeks and the sustained virological response (SVR) rate was only 45% for genotype 1, [6, 7], the most common genotype in Montréal [8]. Moreover, this treatment regimen required weekly injections of peginterferon, and was associated with severe adverse events such as rash, anemia and/or depression [6, 7]. The current HCV cascade of care is largely reflective of the period where these treatments represented the only option to cure HCV.

However, since 2014 direct-acting antiviral (DAA) molecules for HCV treatment are increasingly available. These treatments are more effective (>90% SVR for all genotypes), shorter (12 weeks), less restrictive as oral regimens, and they cause few or no adverse events [9–14]. These recent advance in the HCV therapeutic area provides an interesting opportunity for the elimination of HCV infection in this population, defined as the ‘reduction to zero of the incidence of infection [15, 16]. The successful treatment of infected individuals could limit the transmission of the virus to current or future injecting partners and prevent the occurrence of serious health outcomes such as end-stage liver disease. However, if a “Treatment as Prevention” (TasP) strategy is to work, it will require enhancements in the “HCV cascade of care”, including increased HCV testing, linkage to HCV care, improved liver fibrosis assessment, greater HCV treatment uptake, and improved adherence and cure of HCV [17].

In this paper, we used a previously developed dynamic stochastic model for HCV transmission in PWID [18] to estimate the impact of a TasP strategy on HCV transmission and related morbidity when varying the components of the HCV cascade of care among PWID in Montréal.

## Methods

Dynamic modeling was used to simulate HCV transmission and natural history. Details about the model and related parameters are provided below and elsewhere [18].

### Model

A previously described model of HCV transmission was used to estimate the impact of a TasP strategy [18]. Briefly, it is a stochastic individual-based model including the social network of PWID (i.e., people who inject together), to take into account the background risk of HCV infection between injecting partners [19] (see Additional file 1: S1).

**Fig. 1:**
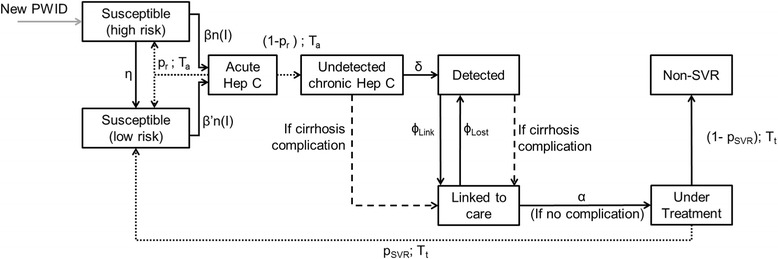
Transition chart of the model for HCV infection and care. New PWID enters the population as “Susceptible (high risk)” – corresponding to recent initiation of injection – for all the simulation period; this assures a constant population size (i.e. each death in the population implies the arrival of a new PWID). Plain arrows correspond to transitions occurring according to exponential probability distributions. Dashed lines correspond to transitions occurring after a fixed time with a given probability. Dotted lines correspond to transitions related to the natural history model. An individual is considered as Detected if he/she has an HCV antibody positive test. An individual is considered as Linked to care if he/she had one or more consultation related to his/her HCV infection in the past 6 months (with the first link to care corresponding to the first positive RNA test, see Additional file 1: S2)

**Fig. 2:**
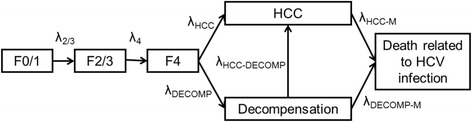
Transition chart for the natural history of chronic hepatitis C. All transitions occur according to exponential probability distributions. Fibrosis progression is quantified using Metavir Score [42]: F0 = No fibrosis, F1 = Portal fibrosis without septa, F2 = portal fibrosis with few septa, F3 = numerous septa without cirrhosis, F4 = cirrhosis. Metavir fibrosis scores F0 and F1 (respectively F2 and F3) were gathered in a F0/1 (respectively F2/3) state

Figure 1 describes the transition chart of the model for HCV transmission and care. Figure 2 presents the natural history of HCV infection; two complications can occur in cirrhosis: decompensation and hepatocellular carcinoma (HCC), which can lead to death. The infection rate for an individual depends on his/her social network, the latter being modelled by a random graph which is assumed to be static (with replacement of dead PWID by new individuals in the network). Finally, the model includes rates of permanent or sustained injection cessation (i.e., relapse is not considered in the model) and general mortality (i.e., non-HCV-related mortality) which also depends on the injecting status (active or inactive, i.e. after cessation of injection) of PWID overtime.

The starting population is Montréal’s active PWID population (i.e., people who injected in the past 6 months), estimated to be 4,000 individuals [2]. Due to limited data regarding the evolution of the population size of PWID in Montréal, the population size is assumed constant in time: each dead PWID is replaced in the model by another non-infected PWID.

### Parameters

Key parameters are presented in Table 1. Where possible we used regional data reflecting the local context. SurvUDI, a bio-behavioural surveillance system for HCV and HIV infections among PWID in Eastern Central Canada and targeting hard-to-reach PWID, provided most of the estimates for model parameters. Eligibility criteria included age 14 years and older, injecting at least once within the past six months, and speaking French or English [3]. Particularly, the contact rate β was fitted by Approximate Bayesian Computation (or ABC) to reproduce, during the first year of simulation, the incidence observed in Montreal for active PWID participating in SurvUDI for the 2010–2013 period, i.e. 22/100PY. ABC is a bayesian method used to infer some parameters of a model without likelihood estimation [20]. Details about the method are provided in Additional file 1: S2. Other parameters were derived from the scientific literature. Additional file 1: S2 provides details and underlying assumptions for the model.

**Table 1:** Key parameters of the model

Parameter	Value	References	
Population size	4,000	[2]	
Average number of injecting partners during the injecting career	12	Derived from [38]	
Initial distribution (HCV infection and cascade of care)			
*Susceptible with high risk (recent initiation of injection)*	10.10%		SurvUDI, 2012–2014, unpublished data
*Susceptible with low risk (experienced PWID)*	36.80%		
*Acute hepatitis C*	0%^a^		
*Non-detected chronic hepatitis C*	8.40%		SurvUDI, 2012–2014, unpublished data
*Detected, non-linked to care chronic hepatitis C*	24.40%		
*Detected and linked to care chronic hepatitis C*	15.30%		
*Under treatment*	0.40%		
*Non-responders after treatment*	4.60%		
Initial distribution in the natural history model			
*F0/F1*	61.1%		(Private communication, J. Bruneau)
*F2/F3*	23.3%		
*F4*	15.6%		
*Decompensated cirrhosis*	0%^a^		
*HCC*	0%^a^		
Infection rate by injecting partner in Susceptible (low risk)	0.025 y^−1^partner^−1^	Fitted by Approximate Bayesian Computation (ABC) to have a 22.1/100 p-y baseline incidence (SurvUDI, 2010–2013)	
Mean time from the end of acute hepatitis C to detection	2.0y	Derived from SurvUDI, 2012–2014, unpublished data	
Mean duration of the high-risk period, i.e. Susceptibles (high risk, recently initiated PWID)	4.0y	[39]	
Mean time before linkage to care	1.7y	Derived from Notifiable Disease Reporting System of the Montréal Public Health Department	
Loss to follow-up rate	10.3%/y	Derived from SurvUDI, 2012–2014, unpublished data	
Treatment initiation rate when linked to care	5%/y	Approximate value derived from SurvUDI, 2012–2014, based on current number of people under treatment (0.4%)	
Treatment: incoming DAAs regimens			
*Duration*	12 weeks		[9–14]
*SVR rate – treatment naive - all genotypes- clinical trials*	90%		
Mean duration of injecting career	9.5y		[43]

*PWID* people who inject drugs; SVR: sustained virological response

^a^Hypothesis

### Outcomes

The outcomes of interest were: occurrence of HCV infection (average incidence and prevalence after 10y) and related morbidity (average number of cirrhosis complications avoided over 10 and 40y). This time horizon for the number of cirrhosis complications was chosen because of the long delay before the occurrence of HCV-related complications. The outcomes were estimated for whole PWID populations (active plus inactive injectors) except incidence, for which only active PWID are at risk of infection. For each outcome, we presented a mean and the associated 95% confidence interval.

In addition, for each scenario, the mean numbers of treatments initiated (and completed unless the individual dies during the treatment) over 40y were estimated.

### Scenarios

Using 8 different scenarios (see Table 2), we estimated the impact of improvements in the HCV cascade of care on HCV occurrence and morbidity in the Montréal PWID population. One thousand epidemic trajectories were simulated to derive the effects of each of the eight following scenarios:

**Table 2:** Description of the 8 scenarios simulated

Scenario	Time to diagnosis (mean)	Time to linkage to care (mean)	Loss to follow-up rate (%/y)	Treatment eligibility	Treatment rate among eligible PWID	%SVR
1 (reference)	2 y	1.7 y	10.2%/y	F0 → F4	5%/y	81%
2	0.5 y	1.7 y	10.2%/y	F0 → F4	5%/y	81%
3	2 y	0.5y	5%/y	F0 → F4	5%/y	81%
4	2 y	1.7 y	10.2%/y	F0 → F4	5%/y	90%
5	2 y	1.7 y	10.2%/y	F0 → F4	10%/y	81%
6	2 y	1.7 y	10.2%/y	F0 → F4	20%/y	81%
7	0.5 y	0.5 y	5%/y	F0 → F4	20%/y	90%
8	0.5 y	0.5 y	5%/y	F2 → F4	100%/y	90%

*PWID* People who inject drugs, *SVR* sustained virological response

S1 (reference): The recommended HCV cascade of care among PWID using Sofosbuvir (for all stages of liver fibrosis), which corresponds to the situation at the time the study was done. Mean time from the end of acute hepatitis C to detection: 2y; mean time from detection to linkage to care: 1.7y; annual loss to follow-up rate: 10.2%/y; treatment initiation rate (when linked to care): 5%/y; SVR rates: 81% (90% for Sofosbuvir based regimens in clinical trials multiplied by a coefficient of 0.9 to account for the difference between real-world and clinical trial contexts, see Additional file 1: S2); duration of the treatment: 12 weeks.

In this reference scenario, treatment initiation can occur for fibrosis scores ≤ F4. Indeed, the initiation of HCV treatment among PWID should be individualized as recommended by several professional societies and expert panels (Canadian Association for the Study of the Liver [21] and the American Association for the Study of the Liver [22]), and current recommendations regarding the use of Sofosbuvir in Québec do not include severe fibrosis scores as a requirement for reimbursement ([23], Québec Institute for Excellence in Health and Social Services (INESSS) [24]). Such restrictions exist, however, for more recent combined DAA regimens [23].

S2: S1 with an improvement in the mean time to detection of chronic HCV from 2y to 0.5y (1y after the infection, due to the 6 months of acute hepatitis C in the model), and corresponding to annual testing, as supported by AASLD guidelines [22].

S3: S1 with an improvement in linkage to care, with a mean time to linkage to care from 1.7y to 0.5y and a loss to follow-up rate from 10.2%/y to 5%/y.

S4: S1 with an improvement in adherence to treatment. In this scenario, we improved the SVR rate of 81% to the level demonstrated in clinical trials, i.e. 90%.

S5: S1 with an improvement in treatment initiation rate from 5%/y to 10%/y when linked to care.

S6: Improvement in treatment initiation rate from 5%/y to 20%/y when linked to care.

S7: Combined scenarios S2, S3, S4 and S6 to determine the impact of improvements in the entire cascade of care.

S8: S7 with an initiation of HCV treatment at fibrosis levels F2-F3-F4 only. Due to the high cost of the new DAAs (55,000$CAD [25]), there may be a reluctance to treat people with minimal fibrosis (F0/F1 fibrosis scores) [22]. Therefore, simulations were performed based on treatment initiation at fibrosis scores between F2 and F4, i.e., 100% of the PWID with moderate or severe fibrosis were treated (vs. 5% of all PWID in S1) while those with F0 and F1 were excluded from treatment. The purpose of this scenario is to assess the optimal outcomes that could be obtained when restricting treatment to more advanced fibrosis in a context where all PWID eligible for treatment are treated.

### Sensitivity analysis

The confidence intervals presented in the main analysis only reflect the uncertainty due to the stochastic processes of the model and not that associated with uncertainty in parameter estimates (see Additional file 1: S6). To assess the impact of the latter on our results, we performed several sensitivity analyses.

First, we performed a deterministic univariate sensitivity analysis by varying the parameter values based on the uncertainty interval (e.g., 95% confidence interval) if available, or by using values from other studies. We varied the following parameters in the model: infection rate, mean duration of the high-risk period, relative risk of reinfection after SVR, time between chronic infection and detection, average time before linkage to care and loss to follow-up rate, mortality rates, average duration of injecting career and all the transitions rates in the natural history model (see Additional file 1: S5). Due to the uncertainty about the number of injecting partners, we also varied this parameter to cover the range of likely values in the literature (between 3 and 15) [18]. Moreover, we did a sensitivity analysis where the spontaneous recovery rate was set at 41% vs. 26%, as in the main analysis, because some studies suggest a higher recovery rate among PWID compared with the general population [26, 27]. Finally, we undertook an analysis using a high-risk period of 1 year (instead of 4 years as done in the main analysis) [28]. The details of theses analyses are provided in Additional file 1: S5.

## Results

**Fig. 3:**
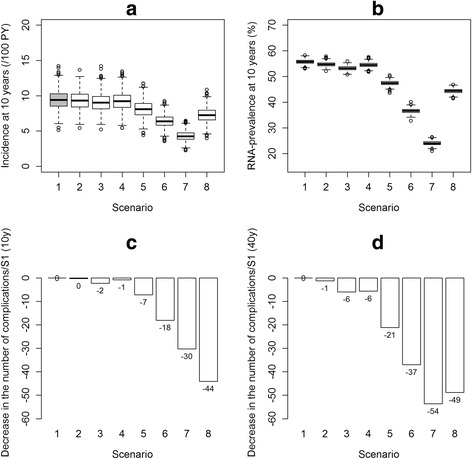
Results according to various HCV cascade of care scenarios; 1,000 simulations. **a**. Boxplots of the incidence at 10 years; **b**. Boxplots of the prevalence at 10 years; **c**. Proportion of cirrhosis complications avoided after 10 years (mean percentage of new cirrhosis complications avoided, compared with the reference scenario (S1)); **d**. Proportion of cirrhosis complications avoided after 40 years (mean percentage of new cirrhosis complications avoided, compared with the reference scenario (S1)). S1 (reference): The current HCV cascade of care using the new DAAs. S2: S1 with an improvement in the mean time to detection of chronic HCV from 2y to 0.5y. S3: S1 with an improvement in linkage to care, with a decrease in mean time to linkage to care from 1.7y to 0.5y and a loss to follow-up rate from 10.2%/y to 5%/y. S4: S1 with an improvement in adherence to treatment, i.e. we improved the SVR rate of 81% to the level demonstrated in clinical trials, i.e. 90%. S5: S1 with an improvement in treatment initiation rate from 5%/y to 10%/y when linked to care. S6: Improvement in the treatment initiation rate from 5%/y to 20%/y when linked to care. S7: Combined scenarios S2, S3, S4 and S6 to determine the impact of improvements in the entire cascade of care; no fibrosis criteria for treatment initiation. S8: S1 with an initiation of HCV treatment at fibrosis levels F2-F3-F4 only

Figure 3 presents boxplots representing the prevalence and incidence distributions after 10y, and the proportion of cirrhosis complications avoided over 10 and 40y, for each scenario compared with S1, the reference. In addition, the reader can refer to Additional file 1: S3, S4 and S7 for information on the evolution of outcomes over time, the impact of each scenario on the disposition of PWID in the cascade of care after 10 years, and the number of infections and HCV-related deaths for each scenario, respectively.

### HCV transmission in the population

In the reference scenario S1, the mean incidence and prevalence estimates after 10y were 9.4/100PY [95% confidence interval: 9.2; 9.7] and 55.8% [55.6; 55.9], respectively. Improved testing in S2, linkage to care in S3 or adherence to treatment in S4, each taken separately, led to similar incidence estimates of 9.3/100PY [9.1; 9.6], 9.1/100PY [8.8; 9.3] and 9.2/100PY [9.0; 9.5], respectively. S2, S3 and S4 also led to similar prevalence estimates: 54.7% [54.6; 54.9], 53.2% [53.1; 53.4] and 54.5% [54.4; 54.7]. Improvements in the treatment initiation rate, from 10%/y to 20%/y in S5 and S6 led to a decrease in HCV occurrence with incidence estimates of 8.1/100PY [7.9; 8.3] and 6.4/100PY [6.2; 6.6], respectively. Similarly, prevalence estimates decreased for S5 and S6: 47.5% [47.3; 47.6] and 36.6% [36.4; 36.7]. The combined scenario S7 (representing improvements in the whole cascade of care) was the most effective with the incidence dropping to 4.3/100PY [4.2; 4.4] and prevalence to 24.0% [23.9; 24.2] after 10y. Finally, when restricting treatment to F2-F4 fibrosis scores in S8, the incidence and prevalence estimates were 7.3/100PY [7.1; 7.5] and 44.3% [44.1; 44.5], respectively.

### Chronic hepatitis C complications

Compared with the reference scenario S1, improved testing in S2, had almost no impact resulting in 0% [−1; 2] and 1% [0; 3] of cirrhosis complications avoided over 10 and 40y, respectively. Improved linkage to care in S3, and adherence to treatment in S4, had moderate effects in the long term, with 2% [1; 4] and 1% [−1; 3] of complications avoided after 10y and 6% [5; 7] and 6% [5; 7] after 40y. Improvements in the treatment initiation rate from 10%/y in S5 and to 20%/y in S6, resulted in the avoidance of 7% [6; 9] and 18% [17; 20] of complications after 10y, respectively, while greater decreases were observed after 40y: 21% [20; 22] and 37% [36; 38]. The combined scenario S7 demonstrated a decrease of 30% [29; 32] after 10y, and 54% [53; 54] after 40y, in the number of cirrhosis complications. Finally, treating only F2-F4 fibrosis levels in S8 led to a decrease of 44% [43; 45] and 49% [48; 50] in complications after 10 and 40y, respectively.

### Sensitivity analysis

The tornado graphs in Additional file 1: S5 present variations in outcomes assuming the conditions of S1 while considering parameter uncertainty levels. The parameters determined to be most sensitive (top 10) in outcome estimation are presented for each outcome. For the incidence after 10 years, the most sensitive parameters were the mean time to cessation of injection (with a variation in the reference scenario S1 of −6.0/100 p.y, +3.9/100 p.y.), the treatment initiation rate (−1.3/100 p.y., 1.7/100 p.y.) and the infection rate per infectious injecting partner (−1.6/100 p.y., 1.1/100 p.y.). The most sensitive parameters for the prevalence after 10 years were the treatment initiation rate (−8.3%, +8.7%) and the mean time to cessation of injection (−9.0%, +5.1%). Finally, for the number of cirrhosis complications within 10 years, estimates were most sensitive to the transition rate from F2/F3 to F4 (−18%, +22%), the fibrosis distribution in the population (−28%, +0%) and the decompensation rate (−10%, +15%). For cirrhosis complications after 40 years, estimates were most sensitive to the following parameters: the treatment initiation rate (−21%, +37%), the transition rate from F2/F3 to F4 (−29%, +26%) and the transition rate from F0/F1 to F2/F3 (−15%; +11%).

In other sensitivity analyses, the trends of our results remained unchanged when we varied the number of injecting partners. In addition, we also simulated the 8 scenarios with the lower and upper bounds of the mean time to cessation of injection used in the univariate sensitivity analysis (4.7 years and 14 years), due to the large impact on prevalence and incidence. The trends observed for the various scenarios were relatively unchanged.

## Discussion

**Fig. 4:**
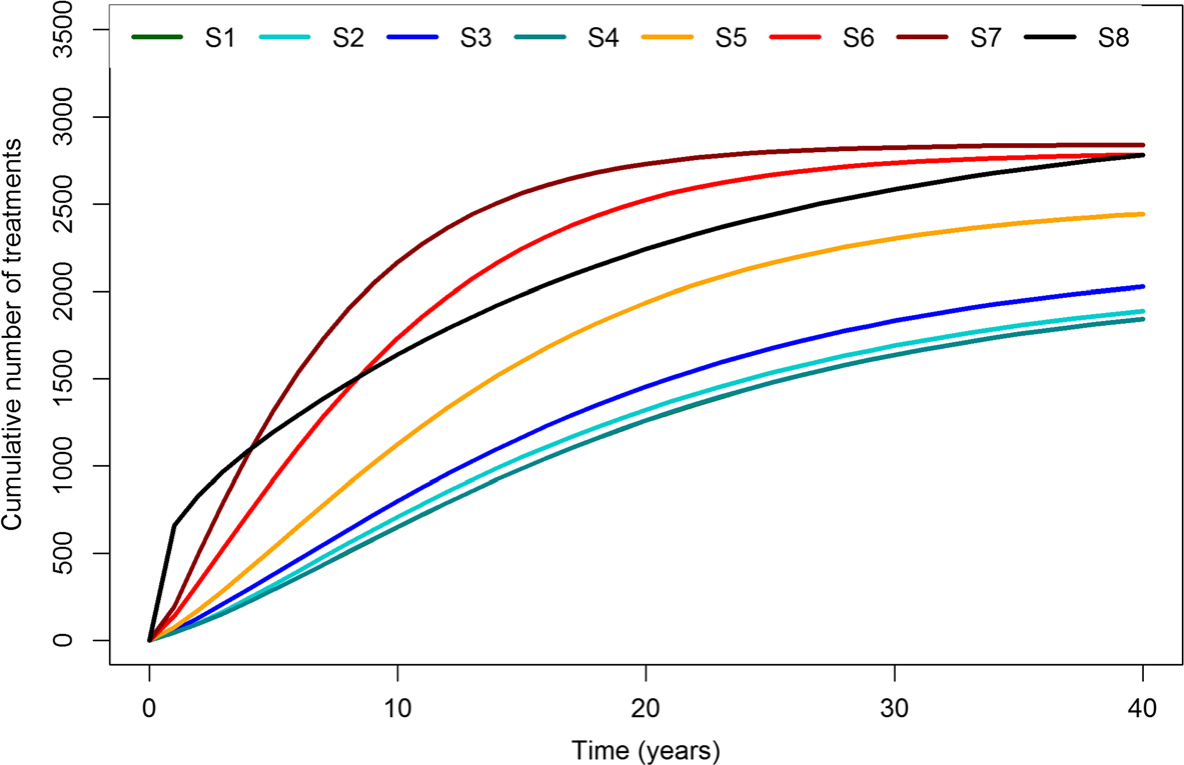
Cumulative number of treatments initiated in each scenario over 40 years of simulation

We used an individual-based model to simulate the evolution of HCV infection among active PWID in Montréal while varying the cascade of care. Model parameters were primarily informed by local data. The results showed, compared with the current cascade of HCV care, that the best approach to curtail ongoing HCV transmission and future cirrhosis complications in this population, is to improve access to treatment. By increasing the treatment initiation rate from 5%/y to 10%/y and 20%/y, prevalence at 10y decreased from 55.8% to 47.5% and 36.6%, respectively. Similarly, incidence rates at 10y dropped from 9.4/100PY to 8.1/100PY and 6.4/100PY, respectively. In addition, the number of cirrhosis complications decreased by 21% and 37% over 40y using 10%/y and 20%/y treatment initiation rates. Conversely, improved testing, linkage to care or adherence to treatment alone, led to minimal decreases in disease burden. However, combining these improvements with a higher treatment initiation rate permitted a decrease of almost 50% in the prevalence and incidence at 10y and the number of cirrhosis complications over 40y. Finally, by restricting treatment to patients with moderate and severe fibrosis (S8), the impact on HCV transmission was considerably lower compared to S7 (no fibrosis restriction, treatment initiation of 20%/y), even in the optimistic case where 100% of the eligible individuals were treated. However, there was a greater impact on the reduction in the number of cirrhosis complications in the short term: −44% (10y). Nevertheless, both scenarios S7 and S8 would require a similar number of treatment courses over 10y; approximately 1,500, see Fig. 4.

These results show, that even in the context of new DAAs, a large decrease in the disease burden using TasP, first requires greater access to treatment for PWID once they are diagnosed and linked to care. When this treatment scale-up is achieved, improvements in other parts of the cascade of care could result in additional benefits for both HCV transmission and morbidity/mortality. Without this treatment scale-up, increased testing or linkage to care would be of limited benefit; these patients would not initiate antiviral treatment before several years, while experiencing ongoing fibrosis progression and continuing to be a source for new HCV infections. Indeed, even if improvements in testing and linkage to care are possible, testing and linkage to care rates are already high relative to the treatment initiation rate, with only 5%/year of PWID newly diagnosed and linked to care initiating antiviral treatment. This represents a small number of individuals, and is reflected in the number of people initiating treatment in scenarios 2 and 3 (Fig. 4).

This approach would be inconsistent with recent statements from the European Association for the Study of the Liver (EASL) [29] where screening of PWID is promoted in part to improve access to treatment, but also to reduce transmission. For the same reason, while treatment initiation restricted to fibrosis scores ≥ F2 would reduce liver related morbidity, it would also delay treatment for many other infected PWID. Indeed, in our study, S7 (where treatment is initiated regardless of fibrosis level and at a rate of 20%/year) and S8 (where treatment is initiated immediately for fibrosis ≥ F2 when diagnosed and linked to care and without restricting treatment number) required the same number of treatments over the simulation period (see Fig. 4), however, S7 had a greater impact on transmission. Delaying treatment initiation until fibrosis scores ≥ F2 would effectively allow for several years of ongoing HCV transmission before individuals reach treatment eligibility, and high levels of sustained HCV incidence. This restriction may be justified as it targets treatment to those most in need in whom liver complications are more imminent. However, from a public health perspective, the treatment of patients in the absence of liver disease (low fibrosis scores) is most important to reduce HCV occurrence, and consequently the disease burden over the long-term.

Modeling studies in other settings showed that even a small increase in treatment availability for PWID can result in a large decrease in HCV transmission in the context of highly effective antivirals [30–32], particularly in a low prevalence context [32]. However, these models did not integrate the entire cascade of care, and thus did not identify the specific steps in the cascade that have the largest impact on the course of the HCV epidemic. In Montréal, this appears to be treatment initiation once PWID are diagnosed and linked to care.

In our sensitivity analysis, the mean time to cessation of injection and the infection rate per infected injecting partner had a strong impact on HCV incidence estimates (see Additional file 1: S5). These results suggest that improvements in primary and secondary prevention interventions aimed at reducing the harms of substance use (e.g., delayed initiation of injection drug use, provision of clean injection equipment, opioid substitution therapies, and supervised injection facilities) would complement a TasP strategy. A previous modeling study in the United Kingdom demonstrated the importance of combining risk reduction measures with a treatment scale-up to achieve a high decrease in HCV prevalence [33]. In our model, the current situation of risk reduction measures in Montréal was implicitly included in the infection rate per infected partner values and the time to cessation of injection; the heterogeneity with respect to harm reduction uptake was neglected. Estimating the impact of these preventive public health strategies, in addition to variations in the HCV cascade of care, would require a more complex model including information on injecting drug use initiation, injection equipment distribution, use of opioid substitution therapies/programs, and supervised injection facilities, expected soon in Montréal [34]. Further investigation is needed to incorporate them in the model.

This study has several limitations. First, the network model is static and relatively simple compared with those for PWID in other countries using chain referral sampling [19]. The paucity of data about the network dynamic and topology constrained us, and the development of a more realistic model would require field studies on PWID networks. Also, for simplicity, the model did not explicitly include other comorbidities common in PWID such as HIV infection [3]. We did not assume a change in risk-taking among PWID after a positive hepatitis C diagnosis. Some published studies show that awareness of HCV infection is not associated with a decrease in risk-taking [35, 36]. While current recommendations promote an individual-based treatment decision for PWID [21, 22], treatment is probably preferentially initiated in PWID with advanced levels of fibrosis. However, in our reference scenario, the treatment initiation is independent of the fibrosis score. Finally, with the high cost of the new DAAs (around 55,000$ Canadian for a 12-week course [25]), extended access to these antivirals for the PWID population would mean increased costs for the health system (see Fig. 4 for the number of completed treatment courses needed for each scenario). Using projections based on the current number of patients treated, the cost of the introduction of the new DAAs for the public health insurance system in Québec is estimated to be 45 million Canadian dollars for the first three years after introduction [24]. In addition, improving testing or linkage to care would also be associated with costs. However, these high treatment costs should be balanced against savings realized by averted cirrhosis complications [37]. Future modeling works could consider including health care costs to estimate the costs of various strategies, including one involving TasP.

Our study also has several strengths. The large amount of local data available through ongoing regional surveillance work (SurvUDI and the Notifiable Disease Reporting System of the Montréal Public Health Department) and numerous past and current epidemiological studies [8, 38–40] ensures the model reflects the current situation of HCV infection and care for PWID in Montréal. Also, the model included the entire cascade of care for chronic hepatitis C with testing, linkage to care and treatment.

## Conclusions

To conclude, TasP could lead to a large decrease in chronic hepatitis C burden among PWID in Montréal. The success of this strategy rests on first expanding access to antiviral treatment to PWID already engaged in HCV care. From a public health perspective, access to antiviral treatment is a priority focus in improving the HCV cascade of care. Limiting treatment to moderate to severe fibrosis, while effective in circumventing cirrhosis complications in the short-term, would do little to curtail ongoing HCV transmission in this population. Coupling greater treatment access with ongoing improvements in the HCV cascade of care would ultimately result in less HCV occurrence and disease burden in Montreal. Regardless, elimination of HCV infection (defined as the reduction to zero of the incidence of infection [15]) in this population would not be expected to occur in the short to mid-term. Such an ambitious objective would require a “TasP+” strategy, which would foster a commitment to greater treatment access as well as harm reduction services. Such a strategy would be in line with the recent recommendations from the Québec Institute for Excellence in Health and Social Services (INESSS): to progressively lower (over several years) the fibrosis threshold for access to combination sofosbuvir + ledipasvir (highly effective, but costly) while also improving harm reductions measures [41]. This would allow Quebec to be the first province in Canada where a true TasP+ programmatic intervention could occur. In future work, a more sophisticated model could help evaluate the impact of a “TasP+” strategy; it would consider ongoing improvements in the HCV cascade of care while also determining the health care investment needed to eliminate HCV infection among PWID.

## Additional files

Additional file 1: Detailed explanation of the model, parameters, sensitivity analyses and additional results. S1: Social network, S2: Model parameters, S3: Evolution of the incidence, prevalence and number of cirrhosis complications per scenario, S4: Distribution in the cascade of care after 10 years, S5: Sensitivity analyses, S6: Width of the confidence intervals, S7: Number of HCV infections and HCV-related deaths (DOCX 766 kb)

## Abbreviations

$CAD: Canadian dollars
ANRS: French Agence Nationale de Recherche sur le Sida et les hépatites virales
DAA: Direct-acting antiviral
EASL: European Association for the Study of the Liver
HCC: Hepatocellular carcinoma
HCV: Hepatitis C virus
HIV: Human immunodeficiency virus
INESS: Québec Institute for Excellence in Health and Social Services
PWID: People who inject drugs
PY: Persons-years
SVR: Sustained virological response
TasP: Treatment as Prevention
