# Supplementary material for: The need for treatment scale-up to impact HCV transmission in people who inject drugs in Montréal, Canada: a modelling study
Source: BMC Infect Dis. 2017 Feb 21;17:162. doi: 10.1186/s12879-017-2256-5 (PMC5320702; doi:10.1186/s12879-017-2256-5)
Supplement: Additional file 1: — Detailed explanation of the model, parameters, sensitivity analyses and additional results. S1: Social network, S2: Model parameters, S3: Evolution of the incidence, prevalence and number of cirrhosis complications per scenario, S4: Distribution in the cascade of care after 10 years, S5: Sensitivity analyses, S6: Width of the confidence intervals, S7: Number of HCV infections and HCV-related deaths (DOCX 766 kb) [file 12879_2017_2256_MOESM1_ESM.docx]

# Additional files

## S1: Social network

The social network of our population was defined as the network of injecting partners (i.e. people who inject together) to take into account the background risk of HCV infection between injecting partners (1). Due to a lack of data about the global topography of this network in Montréal, we used an Erdös-Rényi model (2), which can be calibrated using data on the individual-centered network. In this model, each dyad of PWID is linked with a constant probability $p$, which can be estimated from the size of the whole population $N$ and the average number of injection partners expected $\bar{d}$ noting that $p=\bar{d}/(N-1)$.

## S2: Model parameters

The parameters necessary for running the model were mainly provided by SurvUDI data, or by the literature. They are presented in Table S1. We preferentially used data from regional studies when available. Hypotheses underlying some parameter values are detailed below. To assess the impact of these hypotheses on our simulations, we performed several sensitivity analyses (see the main text and Additional file S5).

*Mean number of injecting partners*: in his PhD thesis (3), De, P. detailed a study about the social network of people who inject drugs (PWID) in Montréal. We estimated the average number of injecting partners as the product of the average number of PWID in the individual-centered network, the average proportion of PWID with whom the individual reported having injected, the turnover rate (by month) of the network and the average length (in months) of the injecting career from (Fazito *et al.* (4)) providing an estimate of 12 injecting partners per PWID.

*Chronic HCV prevalence and initial distribution of susceptible PWID*: according to SurvUDI data, 72% of active (in the last 6 months) PWID in Montréal are HCV antibody positive, and based on the proportion of antibody positive individuals among whom RNA can be detected (5), the initial prevalence of chronic hepatitis C is 53%.

*Initial number of acute hepatitis C infections in the PWID population*: due to short duration of acute hepatitis (6 months), we assumed that the baseline proportion of active PWID with an acute hepatitis C infection was negligible.

*Initial distribution in the natural history model*: due to the lack of data about this parameter, we used the distribution of patients followed at the Centre hopitalier de l’université de Montréal (CHUM) for their chronic hepatitis C infection and reporting inject drug use (private communication, J. Bruneau).

*Infection rate by injecting partner:* this rate was fitted to obtain an incidence of 22.1/100 person-years (p.y) during the first year of simulation in the reference scenario. This value corresponds to the estimate found in SurvUDI for the 2010-2013 period. The method used was Approximate Bayesian Computation (6).

Briefly, the main idea of ABC is to fit the (possibly a set of) parameter(s) $\theta$ of a model thanks to simulations and computation of a (possibly set of) statistic(s) $s_{i}, i=1,\ldots,N$ that are compared to the observed values on the data $s_{obs}$. More precisely, we draw a model parameter sample $\theta_{i}, i=1,\ldots,N$ in a prior probability distribution. The model simulations obtained with these parameters are used to obtain the corresponding simulated statistics $s_{i}, i=1,\ldots,N$. Each parameter value is then weighted by $W_{i}=K_{\delta}\left( s_{i}-s_{obs} \right)$, where $K_{\delta}$ is a smoothing kernel with tolerance threshold $\delta$. The weighted sample ${(\theta}_{i}, W_{i}/\sum_{i=1}^{N} W_{i}), i=1,\ldots,N$ gives the posterior probability distribution. We used a variant of the ABC algorithm with linear adjustement to correct $\theta_{i}$ given the other simulations: supposing a linear relation between $\theta$ and $S$, each $\theta_{i}$ is replaced by $\theta_{i}^{*}=\theta_{i}- b(s_{i}-s_{obs})$, with $b$ estimated by linear regression. This variant allows for a tighter posterior distribution. For more details about ABC, the reader can refer to (6, 7).

For our model, we estimated the contact rate β, using a log-transformed uniform prior distribution on [0.01, 0.1], and the incidence observed in SurvUDI survey (22.1/100PY) as target statistic. We performed 25,000 simulations of the model with a tolerance threshold corresponding to 10% of the total number of simulations. Prior and posterior distribution, as well as prior and posterior incidence, are presented in the Figure below.

A.


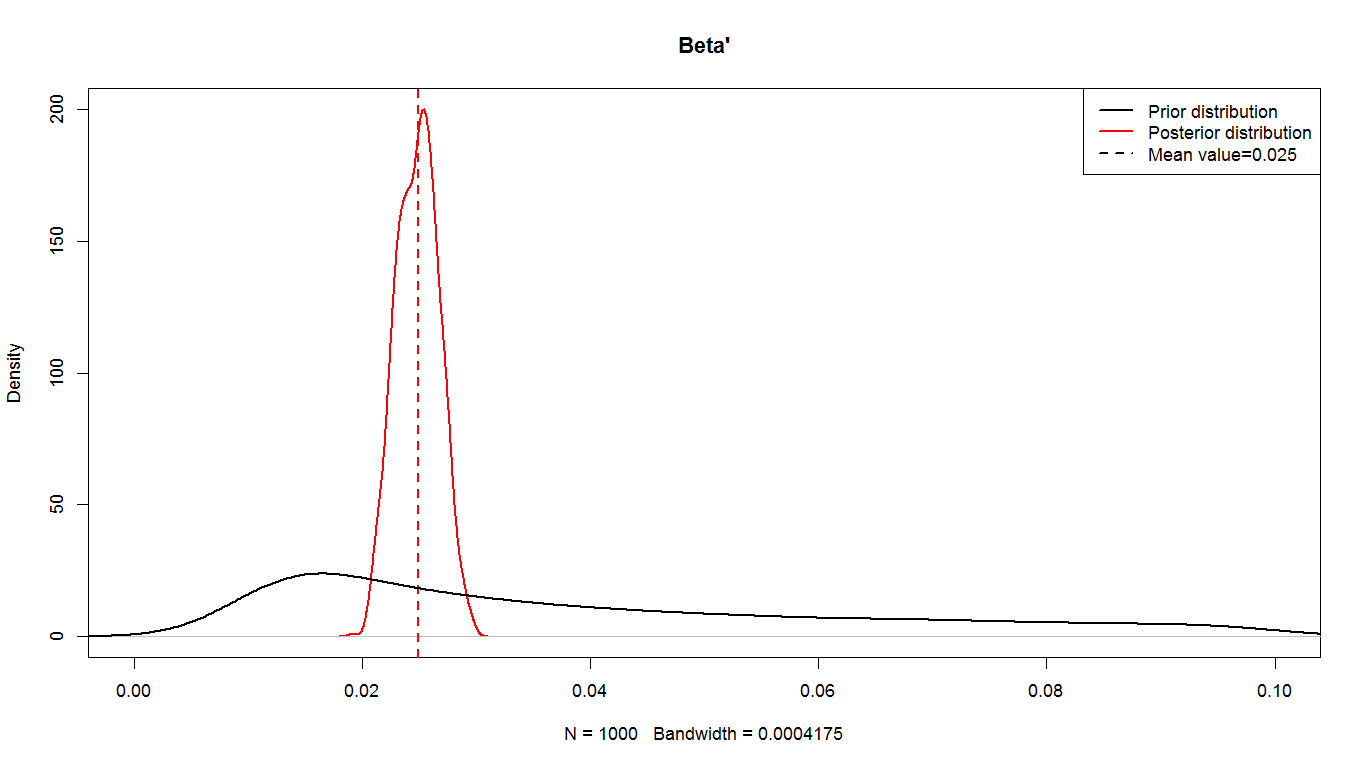


B.


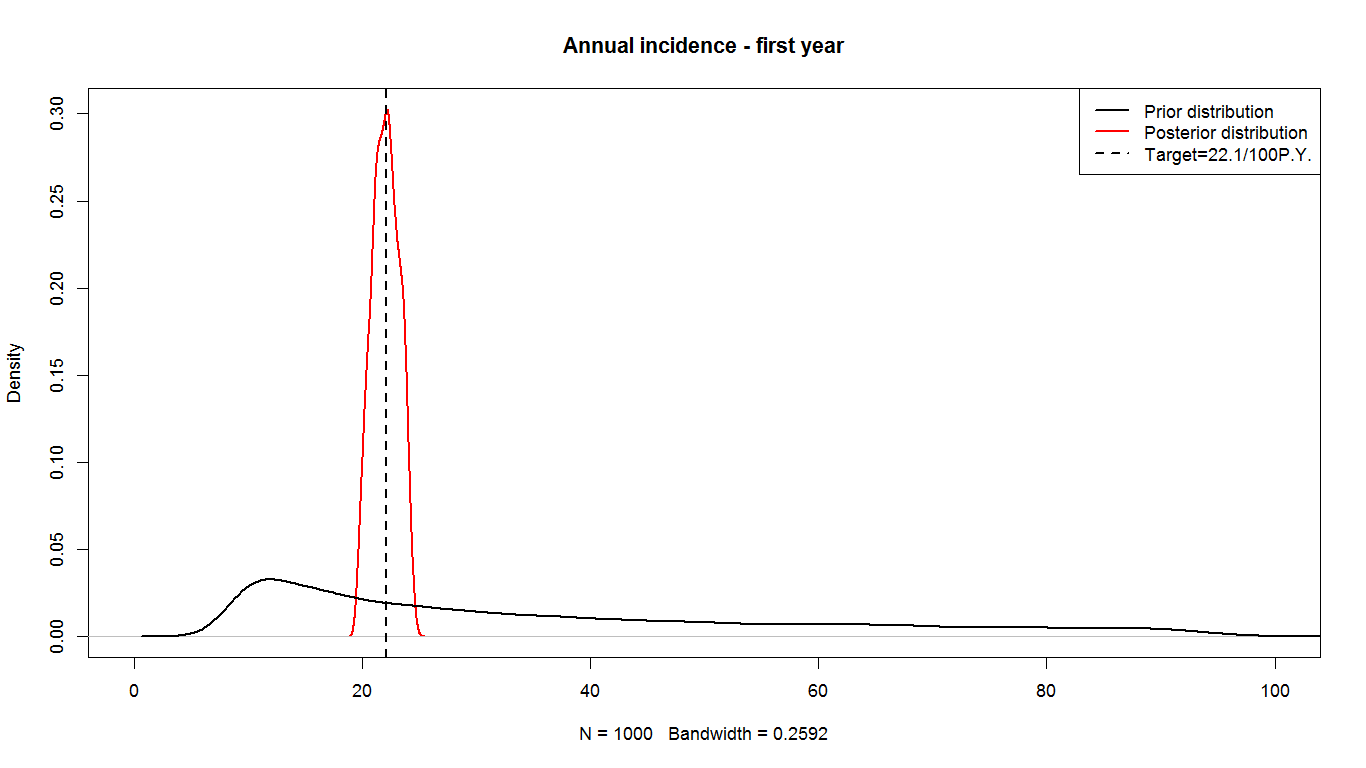


**Figure S1** Prior and posterior distribution for the infection rate per injecting partner and the annual incidence in ABC estimation.

This parameter was also fitted to correspond to changes in the initial set of parameters (mean number of injecting partners and the mean duration before the cessation of injection) in the sensitivity analyses (Additional file S5).

*Mean duration of the high risk period after injection initiation, relative risk of infection during this period and mortality rates of active PWID*: we used estimates based on PWID in studies among street youth of Montréal (8).

*Mean time from the end of acute hepatitis C infection to detection:* this time was derived from the time of the last test in SurvUDI for the 2012-2014 period. The details of the method are given in (9).

*Mean time to linkage to care*: we assumed that after detection, the first consultation for an individual related to his/her HCV infection is measured by the occurrence of a HCV RNA-test. It occurs after a mean duration of 1.7 years according to Notifiable Disease Reporting System of the Montréal Public Health Department.

*Loss to follow-up rate*: according to SurvUDI data, 10.2% of the PWID detected have seen a physician for hepatitis C infection in the past year. Due to our definition of linkage to care (a consultation in the previous 6 months), we considered that these PWID were lost to follow-up during the year, and thus we estimated the annual loss to follow-up rate to 10.2%/y.

*Treatment initiation rate when linked to care:* using SurvUDI data, the current proportion of PWID under treatment at the time of the study was estimated to be 0.4% during the 2012-2014 period; this estimate assumes this proportion remains stable over the short term. The standard of care during this time period was the peg-interferon + ribavirin and usual treatment durations were between 24 and 48 weeks (10). We assumed a duration of 48 weeks for most of the PWID because of the high proportion of hard-to-treat genotypes by dual-therapy in Montreal (around 70% (11)).

If we assume that the 0.4% of individual under treatment and that the time between initiation of treatment and the present time is uniformly distributed among them, we can assume that after 24 weeks (so around 0.5 years) every user will be treated

During this time, 0.4% of the total PWID population should progress from the “Hepatitis C diagnosed, non-linked to care” state (15.3% of the population initially) to under treatment.

Thus, if we note the rate r, we have 0.5×α×15.3=0.4, which gives α =0.052/ year

The corresponding annual probability to initiate treatment is thus p=1-exp(- α)=5%/year.

*Mortality of inactive PWID*: due to the lack of data, we used mortality of the general population in Québec (12).

*Ratio of the effectiveness in the real-world situation to the efficacy in clinical trials:* in absence of data about the effectiveness of new DAAs after approval and market availability, we estimated this ratio based on dual-therapy peg-interferon + ribavirin. We used the following sustained virological response (SVR) rates as clinical trials values: 50% for genotypes 1/4 and 80% for genotypes 2/3 (10); and as real-world values for PWID: 42.9% for genotypes 1/4 and 73.1% for genotypes 2/3 (13). Using the genotype distribution of (11), we estimated this ratio to be 0.90.

*Mean duration before the cessation of injection*: due to the absence of data about PWID in Montréal, we used estimates from Fazito *et al.* for North America (4).

**Table S1** parameters of the model

| **Parameter** | **Value** | **References** | |
| --- | --- | --- | --- |
| Population size | 4,000 | (14) | |
| Average number of injecting partners during the injecting career | 12 | Derived from (3) | |
| Initial distribution (HCV infection and cascade of care) |  |  |  |
| *Susceptible with high risk (recent initiation to injection)* | 10.10% | $\left. \begin{aligned} \\ \\ \end{aligned} \right\}$ | SurvUDI, 2012-2014, unpublished data |
| *Susceptible with low risk (experienced PWID)* | 36.80% |  |  |
| *Acute hepatitis C* | 0%^*^ |  | |
| *Non-detected chronic hepatitis C* | 8.40% | $\left. \begin{aligned} \\ \\ \\ \\ \\ \\ \\ \\ \end{aligned} \right\}$ | SurvUDI, 2012-2014, unpublished data |
| *Detected, non-linked to care chronic hepatitis C* | 24.40% |  |  |
| *Detected and linked to care chronic hepatitis C* | 15.30% |  |  |
| *Under treatment* | 0.40% |  |  |
| *Non-responders after treatment* | 4.60% |  |  |
| Initial distribution in the natural history model |  |  |  |
| *F0/F1* | 61.1% | $\left. \begin{aligned} \\ \\ \\ \\ \end{aligned} \right\}$ | (Private communication, J. Bruneau) |
| *F2/F3* | 23.3% |  |  |
| *F4* | 15.6% |  |  |
| *Decompensated cirrhosis* | 0%^*^ |  | |
| *HCC* | 0%^*^ |  |  |
| Infection rate by injecting partner in Susceptible (low risk) | 0.025 y^-1^partner^-1^ | Fitted by ABC to have a 22.1/100 p-y baseline incidence (SurvUDI, 2010-2013) | |
| Relative risk of infection in Susceptibles (high risk, with recent initiation of injection) | 3 | $\left. \begin{aligned} \\ \\ \end{aligned} \right\}$ | (8) |
| Mean duration of the high-risk period, i.e. Susceptibles (high risk, with recent initiation to injection) | 4 y |  |  |
| Mean duration of acute hepatitis C | 0.5 y | $\left. \begin{aligned} \\ \\ \end{aligned} \right\}$ | (15) |
| Probability of spontaneous recovery | 26% |  |  |
| Mean time from the end of acute hepatitis C to detection | 2.0y | Derived from SurvUDI, 2012-2014, unpublished data | |
| Mean time before linkage to care | 1.7y | Derived from Notifiable Disease Reporting System of the Montréal Public Health Department | |
| Loss to follow-up rate | 10.3%/y | Derived from SurvUDI, 2012-2014, unpublished data | |
| Treatment initiation rate when linked to care | 5%/y | Approximate value derived from SurvUDI, 2012-2014, based on current number of people under treatment (0.4%) | |
| Treatment: incoming DAAs regimens |  |  | |
| *Duration* | 12 weeks | $\left. \begin{aligned} \\ \\ \end{aligned} \right\}$ | (16-21) |
| *SVR rate – treatment naive - all genotypes- clinical trials* | 90% |  |  |
| Annual mortality among active PWID | 18.4/1000 | (22) | |
| Annual mortality among inactive PWID | 7.5/1000 | (12) | |
| Ratio of the effectiveness in real life to the efficacy in clinical trials | 0.90 | Derived from (10, 11, 13) | |
| Mean duration before the cessation of injection | 9.5y | (4) | |
| Transition rate from F0/F1to F2/F3 | 0.052/y | $\left. \begin{aligned} \\ \\ \end{aligned} \right\}$ | (23) |
| Transition rate from F2/F3 to F4 | 0.054/y |  |  |
| Transition rate from F4 to Decompensated cirrhosis | 0.04/y | $\left. \begin{aligned} \\ \\ \\ \\ \\ \\ \\ \\ \end{aligned} \right\}$ | (24, 25) |
| Transition rate from F4 to HCC | 0.021/y |  |  |
| Transition rate from Decompensated cirrhosis to Death related to HCV | 0.306/y |  |  |
| Transition rate from HCC to Death related to HCV | 0.433/y |  |  |
| Transition rate from Decompensated cirrhosis to HCC | 0.21/y |  |  |
| Relative risk after a SVR |  |  |  |
| *Decompensated cirrhosis* | 0.08 | $\left. \begin{aligned} \\ \\ \end{aligned} \right\}$ | (26) |
| *HCC* | 0.27 |  |  |

^*^Hypothesis

PWID: people who inject drugs; SVR: sustained virological response; HCC: hepatocellular carcinoma

## S3: Evolution of the incidence, prevalence and number of cirrhosis complications per scenario

| A.  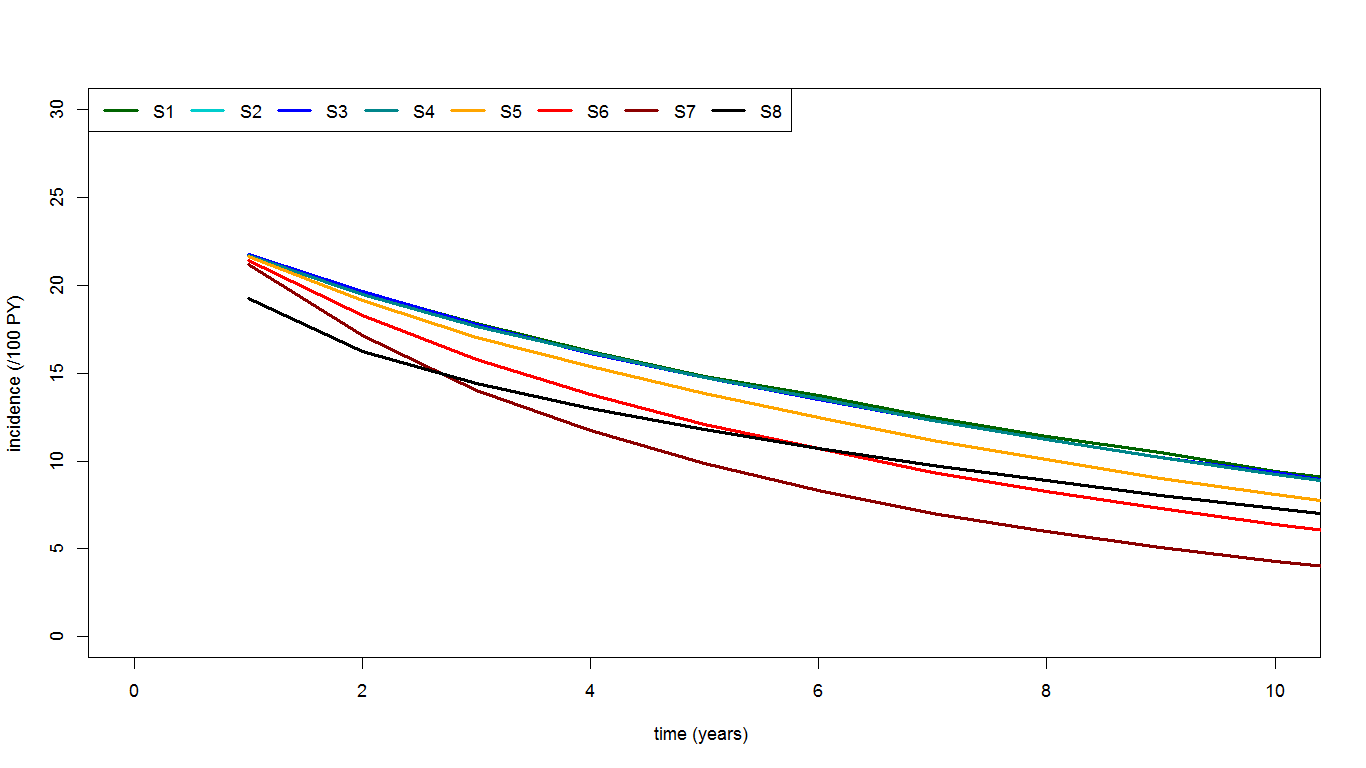 |
| --- |
| B.  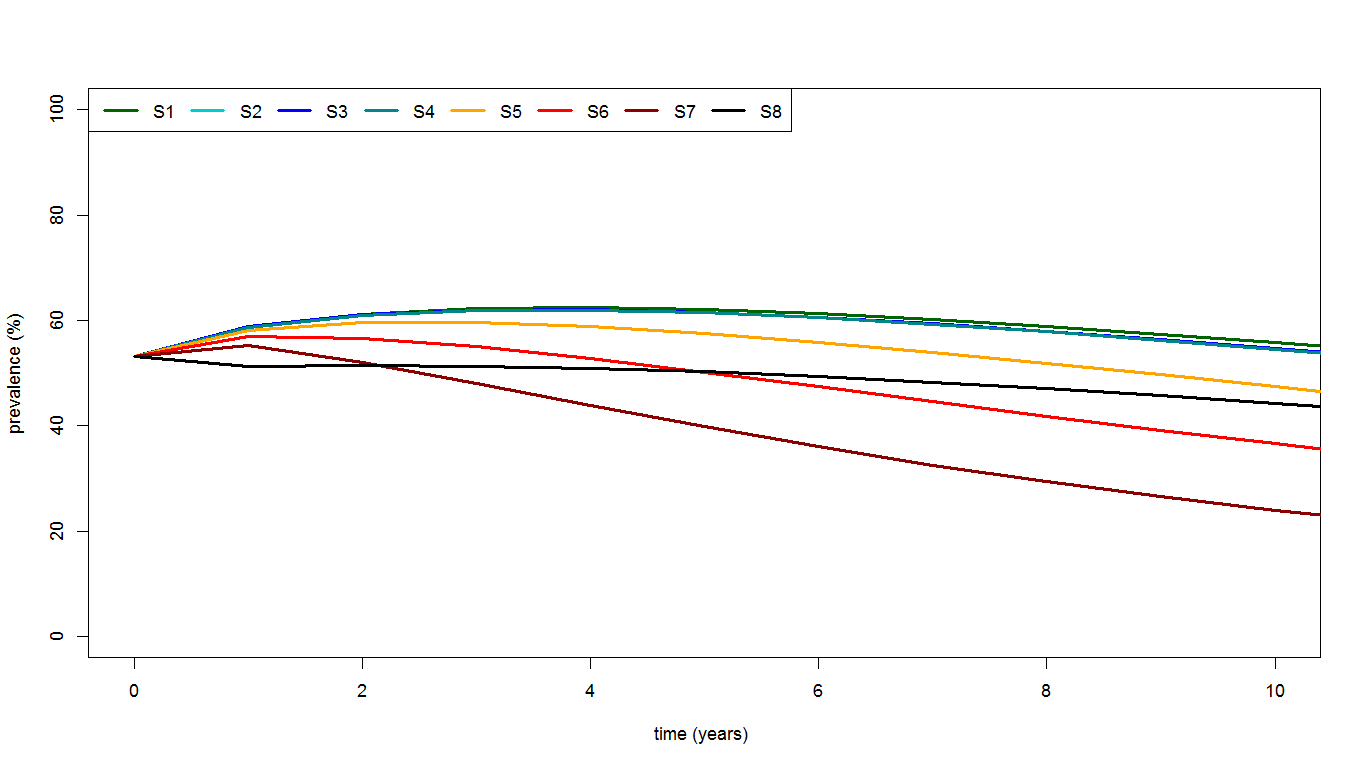 |

| C.  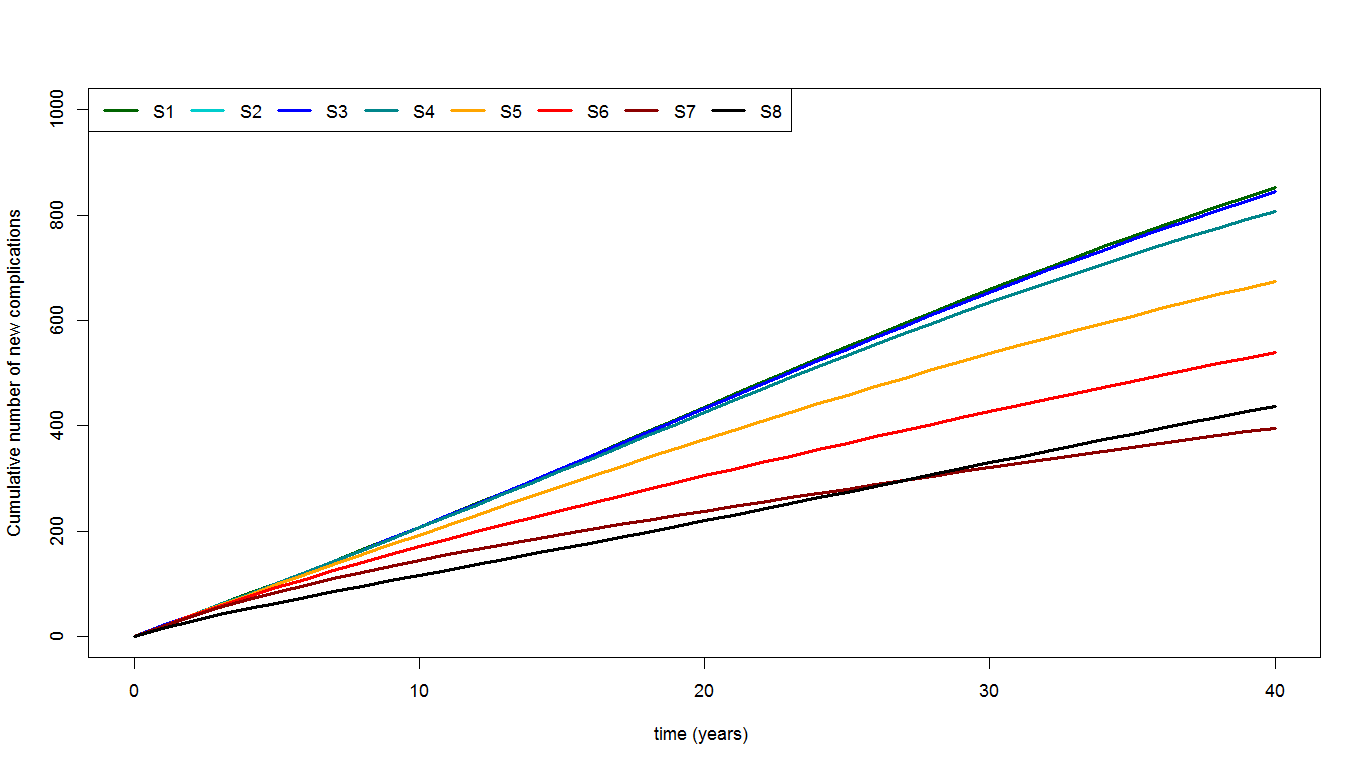 |
| --- |

**Figure S2** Evolution of A) the incidence of HCV in the population over the first 10 years, B) the prevalence of HCV infection over the first 10 years and C) the number of new cirrhosis complications over the first 40 years.

## S4: Distribution in the cascade of care after 10 years


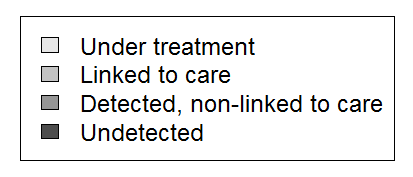


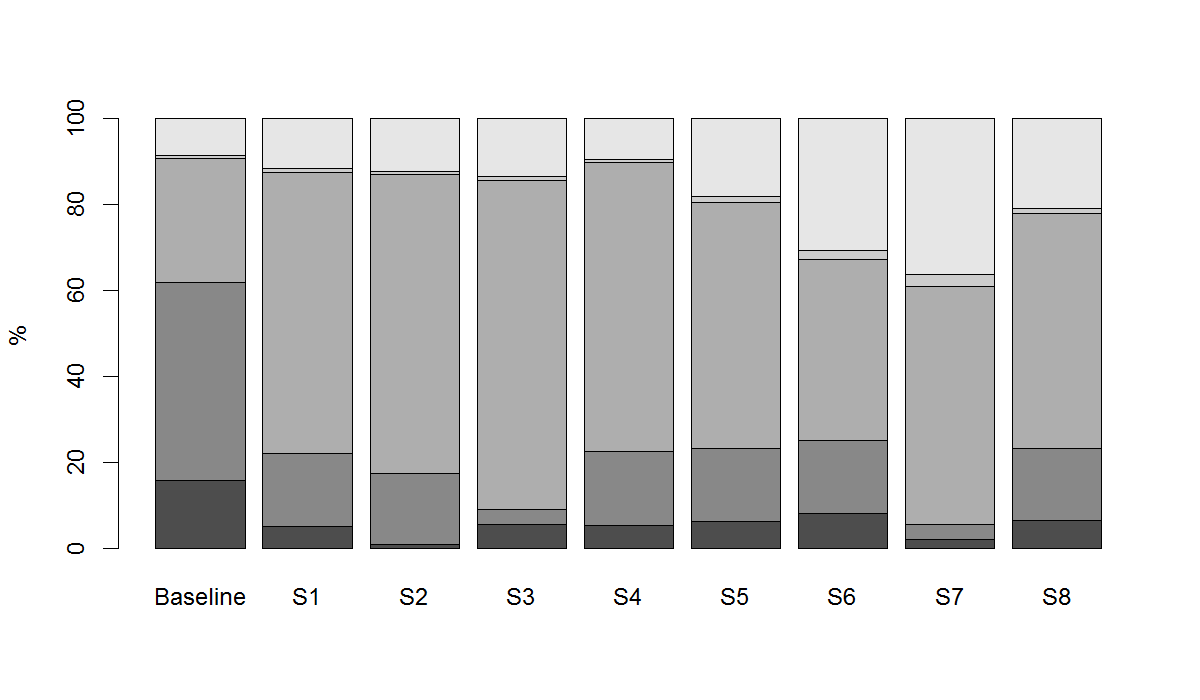


**Figure S3** Distribution of the PWID population in the HCV cascade of care at baseline and after 10 years in the 8 scenarios. S1 (reference): current cascade of care with the new DAAs; S2: improvement in testing; S3: improvement in linkage to care; S4: improvement in adherence to treatment; S5: moderate improvement in the treatment initiation rate; S6: high improvement in the treatment initiation rate; S7: combined S2, S3, S4 and S6; S8: systematic treatment initiation when linked to care, but only for F2-F3-F4 fibrosis scores.

## S5: Sensitivity analyses

### Univariate sensitivity analysis – parameters ranges

In the univariate sensitivity analysis, the values of selected parameters were varied based on their uncertainty intervals (see Table S2). When unavailable, we used values from other settings or assumptions. Explanations for some of these assumptions are given below.

Initial fibrosis distribution in infected PWID was estimated for people infected by drug injection, but not necessarily for those who were active injectors. It also assumes a first evaluation has been done and thus a population that may be more advanced relative to HCV care access. We made this initial distribution vary using less severe fibrosis scores.

In our main analysis, the risk of infection per infectious partner was assumed to remain the same after a SVR. Due to the possible cessation of drug injection, the possible treatment of infectious partners during the time elapsed before treatment, and the high likelihood for PWID who have been treated to have progressed from the high- to the low-risk group, the reinfection rate is actually lower than the primary infection rate in our model: over the first 10 years of simulations, the incidence of primary infection in the reference scenario is 16.0/100 p.y. meanwhile the incidence of reinfection after a SVR is 4.9/100 p.y. However, in the literature, the annual reinfection rate ranges between 2% and 4% (13). Despite the conservative nature of our estimate, we used a relative risk of 0.5 after a SVR in the sensitivity analysis.

**Table S2** Values of the parameters used in the univariate sensitivity analysis

| **Parameters** | **Base case value** | | **Range in sensitivity analysis** | **References** | | |
| --- | --- | --- | --- | --- | --- | --- |
| Initial distribution in the natural history model |  |  | | | |  |
| *F0/F1* | 61.1% | | 75^*^ |  |  | |
| *F2/F3* | 23.3% | | 15^*^ |  |  |  |
| *F4* | 15.6% | | 10^*^ |  |  |  |
|  |  | |  |  |  | |
| Infection rate (per infectious injecting partner) among Susceptibles (low risk) | 0.025/y | | 0.022 – 0.027 |  | From ABC estimation | |
| Mean duration of the high-risk period, i.e. Susceptibles (high risk, with recent initiation of injection) | 4.0 y | | 1.0 -4.0 |  | (27) | |
| Relative risk of reinfection after SVR | 1 | | 0.5-1^*^ |  |  | |
| Time between chronic infection and detection | 2.0 years | | 0.5 – 7.8 |  | (28) | |
| Average time before linkage to care / Loss to follow-up rate | 1.7 y / 14%/y | | 0.5 – 4 / 2.6 – 15 |  | (9) | |
| Annual mortality among active PWID | 18.4/1000 | | 13.8 – 23.8 |  | (29, 30) | |
| Annual mortality among inactive PWID | 7.5/1000 | | 7.0 - 8.0^*^ |  |  | |
| Average duration of injecting career | 9.5 years | | 4.7 - 14 |  | (4) | |
| Transition rate from F0/F1 to F2/F3 | 0.052/y | | 0.031 - 0.074 | $\left. \begin{aligned} \\ \\ \\ \end{aligned} \right\}$ | (23) | |
| Transition rate from F2/F3 to F4 | 0.054/y | | 0.025 – 0.101 |  |  |  |
| Transition rate from F4 to Decompensated cirrhosis | 0.04/y | | 0.032 – 0.052 | $\left. \begin{aligned} \\ \\ \\ \\ \\ \\ \\ \\ \end{aligned} \right\}$ | (24, 25) | |
| Transition rate from F4 to HCC | 0.021/y | | 0.017 – 0.028 |  |  |  |
| Transition rate from Decompensated cirrhosis to Death related to HCV | 0.306/y | | 0.129 – 0.395 |  |  |  |
| Transition rate from HCC to Death related to HCV | 0.433/y | | 0.319 – 0.499 |  |  |  |
| Transition rate from Decompensated cirrhosis to HCC | 0.021/y | | 0.017 – 0.028 |  |  |  |

^*^Hypothesis

PWID: people who inject drugs; SVR: sustained virological response; HCC: hepatocellular carcinoma

The tornado graphs in Figure S4 present variations in outcomes under the conditions of S1 while considering parameter uncertainty levels. The parameters most sensitive (top 10) in outcome estimation are presented for each outcome. Estimates of incidence after 10 years were more sensitive to the mean time to cessation of injection (with a variation in the reference scenario S1 of -6.0/100 p.y, +3.9/100 p.y.), the treatment initiation rate (-1.3/100 p.y., 1.7/100 p.y.) and the infection rate per infectious injecting partner (-1.6/100 p.y., 1.1/100 p.y.). Estimates of prevalence after 10 years were more sensitive to the treatment initiation rate (-8.3%, +8.7%) and the mean time to cessation of injection (-9.0%, +5.1%). Finally, for the number of cirrhosis complications within 10 years, estimates were most sensitive to the transition rate from F2/F3 to F4 (-18%, +22%), the fibrosis distribution in the population (-28%, +0%) and the decompensation rate (-10%, +15%). For cirrhosis complications after 40 years, estimates were most sensitive to the following parameters: the treatment initiation rate (-21%, +37%), the transition rate from F2/F3 to F4 (-29%, +26%) and the transition rate from F0/F1 to F2/F3 (-15%; +11%).

Results of the other sensitivity analyses are presented in Figures S3 to S6. The trends of our results remained unchanged when we varied the number of injecting partners. In addition, we also simulated the 8 scenarios with the lower and upper bounds of the mean time to cessation of injection used in the univariate sensitivity analysis (4.7 years and 14 years), due to the large impact on prevalence and incidence. The trends observed for the various scenarios were relatively unchanged.

**
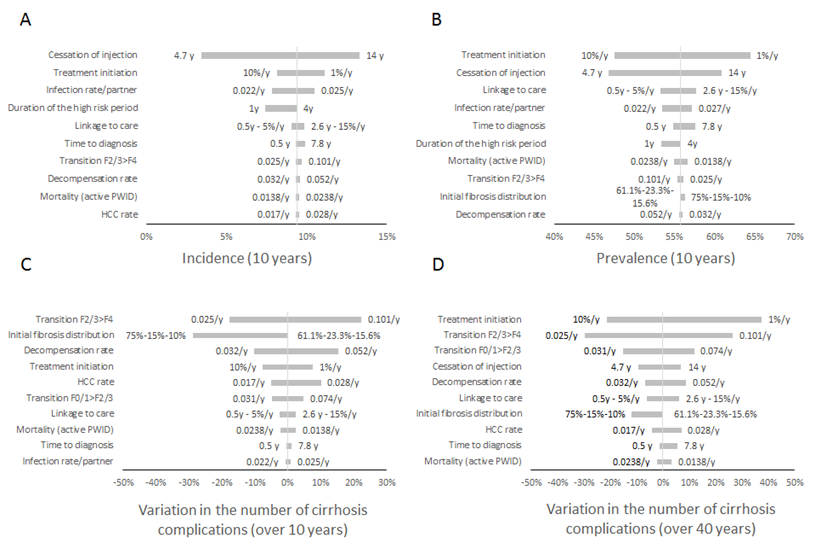
**

**Figure S4** Tornado graphs representing variations in outcomes while considering parameter uncertainty demonstrating the top 10 most sensitive parameters in the model (using the reference scenario (S1)). The corresponding parameters values are given on the charts. A. Incidence at 10 years; B. Prevalence at 10 years; C. Variation in the proportion of new cirrhosis complications over 10 years, compared with the reference scenario (S1); D. Variation in the proportion of new cirrhosis complications over 40 years, compared with the reference scenario (S1).

### Mean number of 3 injecting partners

**
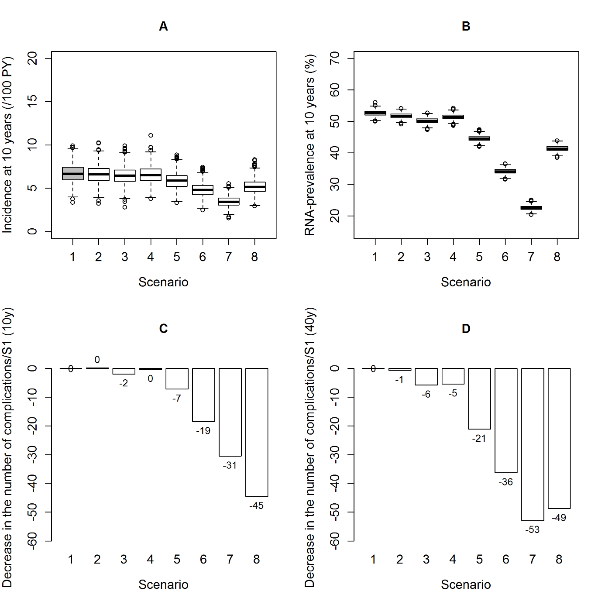
**

**Figure S5** Results according to scenario based on 1,000 simulations, with a mean number of 3 injecting partners. A. Boxplots of the incidence at 10 years; B. Boxplots of the prevalence at 10 years; C. Proportion of cirrhosis complications avoided after 10 years (mean percentage of new cirrhosis complications avoided, compared with the reference scenario (S1)); D. Proportion of cirrhosis complications avoided after 40 years (mean percentage of new cirrhosis complications avoided, compared with the reference scenario (S1)). The infection rate per infectious partner with this set of parameters was estimated to be 0.098/y using Approximate Bayesian Computation to have an initial incidence of 22.1/100 p.y. S1 (reference): The current HCV cascade of care using the new DAAs. S2: S1 with an improvement in the mean time to detection of chronic HCV from 2y to 0.5y. S3: S1 with an improvement in linkage to care, with a decrease in mean time to linkage to care from 1.7y to 0.5y and a loss to follow-up rate from 10.2%/y to 5%/y. S4: S1 with an improvement in adherence to treatment, i.e. we improved the SVR rate of 81% to the level demonstrated in clinical trials, i.e. 90%. S5: S1 with an improvement in treatment initiation rate from 5%/y to 10%/y when linked to care. S6: Improvement in the treatment initiation rate from 5%/y to 20%/y when linked to care. S7: Combined scenarios S2, S3, S4 and S6 to determine the impact of improvements in the entire cascade of care; no fibrosis criteria for treatment initiation. S8: S1 with an initiation of HCV treatment at fibrosis levels F2-F3-F4 only.

### Mean number of 15 injecting partner


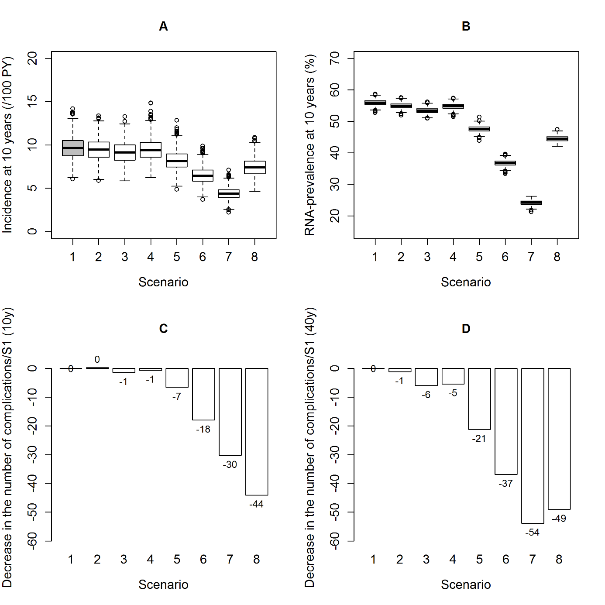


**Figure S6** Results according to scenario based on 1,000 simulations, with a mean number of 15 injecting partners. A. Boxplots of the incidence at 10 years; B. Boxplots of the prevalence at 10 years; C. Proportion of cirrhosis complications avoided after 10 years (mean percentage of new cirrhosis complications avoided, compared with the reference scenario (S1)); D. Proportion of cirrhosis complications avoided after 40 years (mean percentage of new cirrhosis complications avoided, compared with the reference scenario (S1)). The infection rate per infectious partner with this set of parameters was estimated to be 0.020/y using Approximate Bayesian Computation to have an initial incidence of 22.1/100 p.y. S1 (reference): The current HCV cascade of care using the new DAAs. S2: S1 with an improvement in the mean time to detection of chronic HCV from 2y to 0.5y. S3: S1 with an improvement in linkage to care, with a decrease in mean time to linkage to care from 1.7y to 0.5y and a loss to follow-up rate from 10.2%/y to 5%/y. S4: S1 with an improvement in adherence to treatment, i.e. we improved the SVR rate of 81% to the level demonstrated in clinical trials, i.e. 90%. S5: S1 with an improvement in treatment initiation rate from 5%/y to 10%/y when linked to care. S6: Improvement in the treatment initiation rate from 5%/y to 20%/y when linked to care. S7: Combined scenarios S2, S3, S4 and S6 to determine the impact of improvements in the entire cascade of care; no fibrosis criteria for treatment initiation. S8: S1 with an initiation of HCV treatment at fibrosis levels F2-F3-F4 only.

### Mean duration before cessation of injection 4.7 years


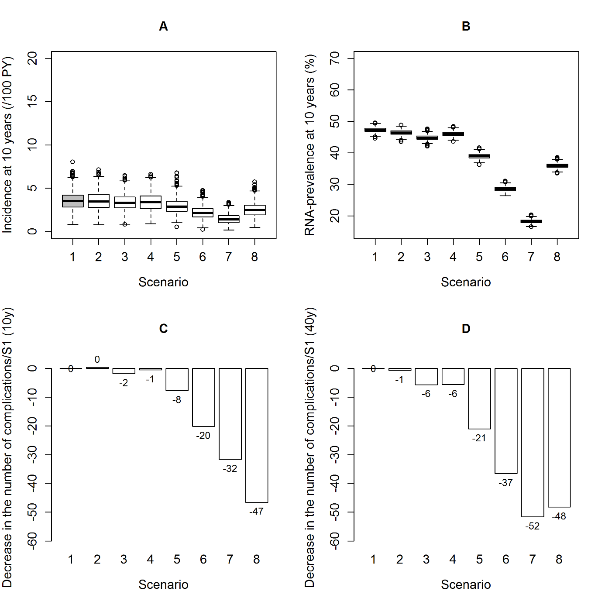


**Figure S7** Results according to scenario based on 1,000 simulations, with a mean duration of the injecting career of 4.7 years. A. Boxplots of the incidence at 10 years; B. Boxplots of the prevalence at 10 years; C. Proportion of cirrhosis complications avoided after 10 years (mean percentage of new cirrhosis complications avoided, compared with the reference scenario (S1)); D. Proportion of cirrhosis complications avoided after 40 years (mean percentage of new cirrhosis complications avoided, compared with the reference scenario (S1)). The infection rate per infectious partner with this set of parameters was estimated to be 0.026/y using Approximate Bayesian Computation to have an initial incidence of 22.1/100 p.y. S1 (reference): The current HCV cascade of care using the new DAAs. S2: S1 with an improvement in the mean time to detection of chronic HCV from 2y to 0.5y. S3: S1 with an improvement in linkage to care, with a decrease in mean time to linkage to care from 1.7y to 0.5y and a loss to follow-up rate from 10.2%/y to 5%/y. S4: S1 with an improvement in adherence to treatment, i.e. we improved the SVR rate of 81% to the level demonstrated in clinical trials, i.e. 90%. S5: S1 with an improvement in treatment initiation rate from 5%/y to 10%/y when linked to care. S6: Improvement in the treatment initiation rate from 5%/y to 20%/y when linked to care. S7: Combined scenarios S2, S3, S4 and S6 to determine the impact of improvements in the entire cascade of care; no fibrosis criteria for treatment initiation. S8: S1 with an initiation of HCV treatment at fibrosis levels F2-F3-F4 only.

### Mean duration before cessation of injection 14 years


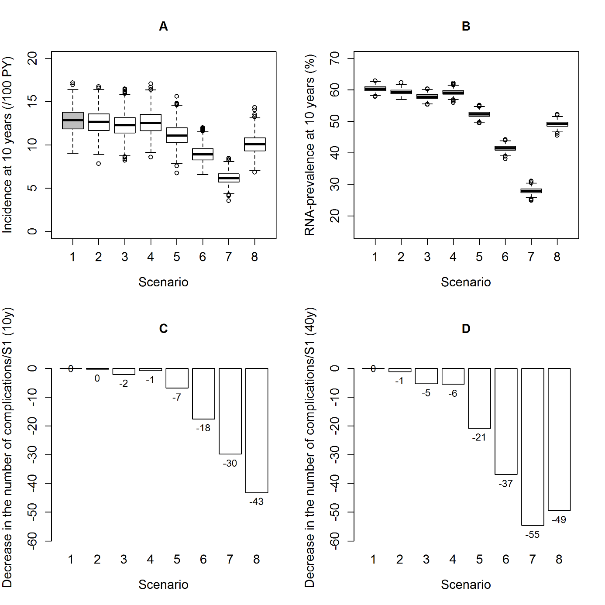


**Figure S8** Results according to scenario based on 1,000 simulations, with a mean duration of the injecting career of 14 years. A. Boxplots of the incidence at 10 years; B. Boxplots of the prevalence at 10 years; C. Proportion of cirrhosis complications avoided after 10 years (mean percentage of new cirrhosis complications avoided, compared with the reference scenario (S1)); D. Proportion of cirrhosis complications avoided after 40 years (mean percentage of new cirrhosis complications avoided, compared with the reference scenario (S1)). The infection rate per infectious partner with this set of parameters was estimated to be 0.024/y using Approximate Bayesian Computation to have an initial incidence of 22.1/100 p.y. S1 (reference): The current HCV cascade of care using the new DAAs. S2: S1 with an improvement in the mean time to detection of chronic HCV from 2y to 0.5y. S3: S1 with an improvement in linkage to care, with a decrease in mean time to linkage to care from 1.7y to 0.5y and a loss to follow-up rate from 10.2%/y to 5%/y. S4: S1 with an improvement in adherence to treatment, i.e. we improved the SVR rate of 81% to the level demonstrated in clinical trials, i.e. 90%. S5: S1 with an improvement in treatment initiation rate from 5%/y to 10%/y when linked to care. S6: Improvement in the treatment initiation rate from 5%/y to 20%/y when linked to care. S7: Combined scenarios S2, S3, S4 and S6 to determine the impact of improvements in the entire cascade of care; no fibrosis criteria for treatment initiation. S8: S1 with an initiation of HCV treatment at fibrosis levels F2-F3-F4 only.

### Spontaneous recovery rate=41%


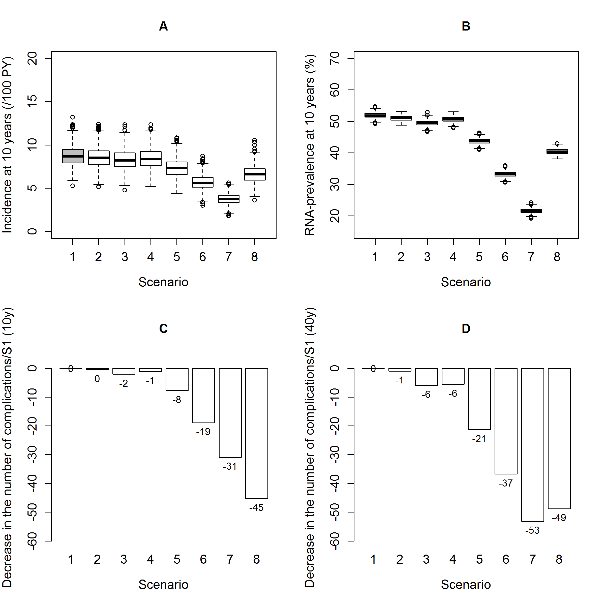


**Figure S9** Results according to a scenario based on 1,000 simulations, with a spontaneous recovery rate of 41%. A. Boxplots of the incidence at 10 years; B. Boxplots of the prevalence at 10 years; C. Proportion of cirrhosis complications avoided after 10 years (mean percentage of new cirrhosis complications avoided, compared with the reference scenario (S1)); D. Proportion of cirrhosis complications avoided after 40 years (mean percentage of new cirrhosis complications avoided, compared with the reference scenario (S1)). The infection rate per infectious partner with this set of parameters was estimated to be 0.024/y using Approximate Bayesian Computation to have an initial incidence of 22.1/100 p.y. S1 (reference): The current HCV cascade of care using the new DAAs. S2: S1 with an improvement in the mean time to detection of chronic HCV from 2y to 0.5y. S3: S1 with an improvement in linkage to care, with a decrease in mean time to linkage to care from 1.7y to 0.5y and a loss to follow-up rate from 10.2%/y to 5%/y. S4: S1 with an improvement in adherence to treatment, i.e. we improved the SVR rate of 81% to the level demonstrated in clinical trials, i.e. 90%. S5: S1 with an improvement in treatment initiation rate from 5%/y to 10%/y when linked to care. S6: Improvement in the treatment initiation rate from 5%/y to 20%/y when linked to care. S7: Combined scenarios S2, S3, S4 and S6 to determine the impact of improvements in the entire cascade of care; no fibrosis criteria for treatment initiation. S8: S1 with an initiation of HCV treatment at fibrosis levels F2-F3-F4 only.

### Duration of the high-risk period=1 year


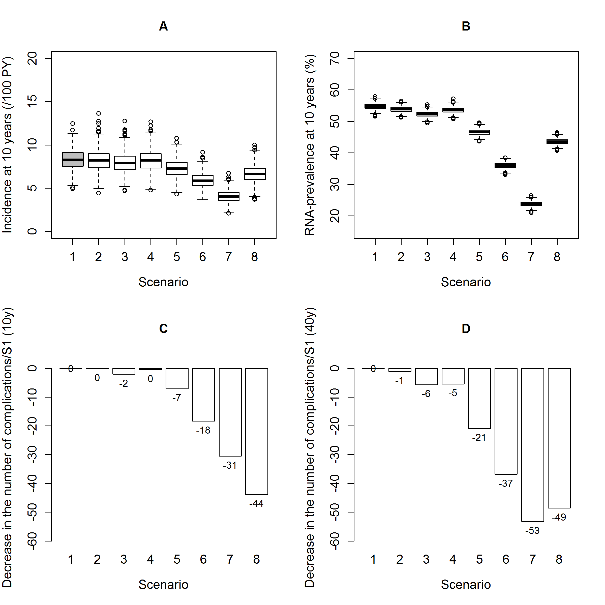


**Figure S10** Results according to scenario based on 1,000 simulations, with the duration of the high risk period=1 year. A. Boxplots of the incidence at 10 years; B. Boxplots of the prevalence at 10 years; C. Proportion of cirrhosis complications avoided after 10 years (mean percentage of new cirrhosis complications avoided, compared with the reference scenario (S1)); D. Proportion of cirrhosis complications avoided after 40 years (mean percentage of new cirrhosis complications avoided, compared with the reference scenario (S1)). The infection rate per infectious partner with this set of parameters was estimated to be 0.027/y using Approximate Bayesian Computation to have an initial incidence of 22.1/100 p.y. S1 (reference): The current HCV cascade of care using the new DAAs. S2: S1 with an improvement in the mean time to detection of chronic HCV from 2y to 0.5y. S3: S1 with an improvement in linkage to care, with a decrease in mean time to linkage to care from 1.7y to 0.5y and a loss to follow-up rate from 10.2%/y to 5%/y. S4: S1 with an improvement in adherence to treatment, i.e. we improved the SVR rate of 81% to the level demonstrated in clinical trials, i.e. 90%. S5: S1 with an improvement in treatment initiation rate from 5%/y to 10%/y when linked to care. S6: Improvement in the treatment initiation rate from 5%/y to 20%/y when linked to care. S7: Combined scenarios S2, S3, S4 and S6 to determine the impact of improvements in the entire cascade of care; no fibrosis criteria for treatment initiation. S8: S1 with an initiation of HCV treatment at fibrosis levels F2-F3-F4 only.

## S6: Width of the Confidence Intervals

Trajectories in a stochastic, individual-based model converge in probability to the solution of a differential equation system when the population size $N$ goes to infinity (31). We can calculate a central limit theorem, which shows the convergence rate is in $1/\sqrt{N}$ (32). Here, $N$=4,000. From this convergence we can deduce asymptotic normality results for the outputs of the model (prevalence, incidence and number of complications) which are simple functions of the epidemic trajectory. Thus, the standard deviation is in $1/\sqrt{N}$.

For our simulations, we did $n$=1 000 Monte-Carlo replications of these outputs. The width of the confidence intervals is thus in $C/\sqrt{n}$, with $C$ a constant. This explains the confidence intervals are tight. These confidence intervals correspond to the set of parameters of the main analysis, they don’t take into account the uncertainty related to these parameters. The latter, which relies on the parameters estimation, was studied in sensitivity analysis.

## S7: number of HCV infections and HCV-related deaths

**Figure S11** Mean decrease in the number of HCV infections after 10 years (A) and 40 years (B), and the mean decrease in the number of HCV-related deaths after 10 years (C) and 40 years (D) relative to the scenario.

*
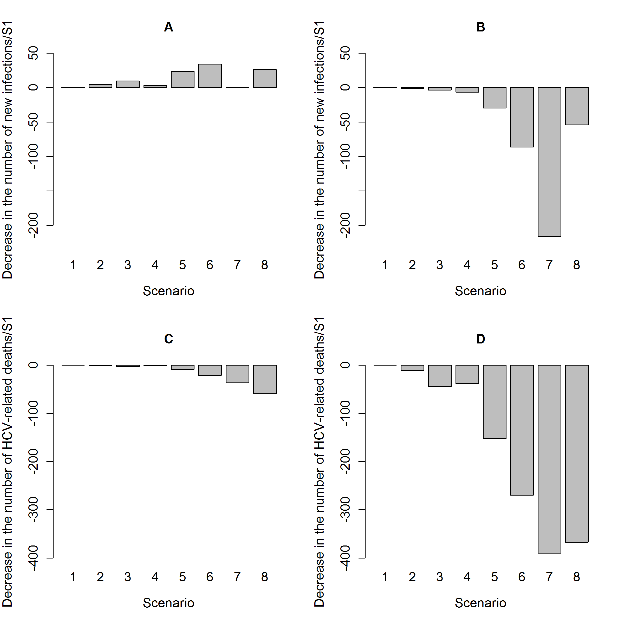
*

Regarding the most notable results, we can see that compared with S1, the number of infections tends to increase after 10 years in all scenarios because of the reinfection of previously treated PWID. In addition, S7 is the most effective scenario after 40 years to decrease both the number of infections and the number of HCV-related deaths.

## References

1. Rolls DA, Daraganova G, Sacks-Davis R, Hellard M, Jenkinson R, McBryde E, et al. Modelling hepatitis C transmission over a social network of injecting drug users. J Theor Biol. 2012;297:73-87.

2. Erdős P, Rényi A. On Random Graphs. I. Publicationes Mathematicae. 1959;6:290–7.

3. De P. Using social networks to better conceptualize risk fro bloodborne viruses among injection drug users. Montréal, Canada: McGill university; 2007.

4. Fazito E, Cuchi P, Mahy M, Brown T. Analysis of duration of risk behaviour for key populations: a literature review. Sex Transm Infect. 2012;88 Suppl 2:i24-32.

5. Trubnikov M, Yan P, Archibald C. Estimated prevalence of Hepatitis C virus infection in Canada, 2011. Canada Communicable Disease Report. 2014;40(19):429.

6. Marin J-M, Pudlo P, Robert CP, Ryder RJ. Approximate Bayesian computational methods. Statistics and Computing. 2012;22(6):1167-80.

7. Blum MG, Tran VC. HIV with contact tracing: a case study in approximate Bayesian computation. Biostatistics. 2010;11(4):644-60.

8. Roy E, Boudreau JF, Boivin JF. Hepatitis C virus incidence among young street-involved IDUs in relation to injection experience. Drug Alcohol Depend. 2009;102(1-3):158-61.

9. Cousien A, Tran VC, Deuffic-Burban S, Jauffret-Roustide M, Dhersin JS, Yazdanpanah Y. Hepatitis C treatment as prevention of viral transmission and liver-related morbidity in persons who inject drugs. Hepatology. 2016;63(4):1090-101.

10. NICE. PegInterferon alfa and ribavirin for the treatment of mild chronic hepatitis C. 2006.

11. Bernier L, Willems B, Delage G, Murphy DG. Identification of numerous hepatitis C virus genotypes in Montreal, Canada. J Clin Microbiol. 1996;34(11):2815-8.

12. Décès et taux de mortalité, Québec, 1900-2014 [Internet]. Institut de la statistique du Québec [cited May 2015]. Available from: http://www.stat.gouv.qc.ca/statistiques/population-demographie/deces-mortalite/301.htm.

13. Aspinall EJ, Corson S, Doyle JS, Grebely J, Hutchinson SJ, Dore GJ, et al. Treatment of hepatitis C virus infection among people who are actively injecting drugs: a systematic review and meta-analysis. Clin Infect Dis. 2013;57 Suppl 2:S80-9.

14. Leclerc P, Vandal AC, Fall A, Bruneau J, Roy E, Brissette S, et al. Estimating the size of the population of persons who inject drugs in the island of Montreal, Canada, using a six-source capture-recapture model. Drug Alcohol Depend. 2014;142:174-80.

15. Micallef JM, Kaldor JM, Dore GJ. Spontaneous viral clearance following acute hepatitis C infection: a systematic review of longitudinal studies. J Viral Hepat. 2006;13(1):34-41.

16. Afdhal N, Reddy KR, Nelson DR, Lawitz E, Gordon SC, Schiff E, et al. Ledipasvir and sofosbuvir for previously treated HCV genotype 1 infection. N Engl J Med. 2014;370(16):1483-93.

17. Afdhal N, Zeuzem S, Kwo P, Chojkier M, Gitlin N, Puoti M, et al. Ledipasvir and sofosbuvir for untreated HCV genotype 1 infection. N Engl J Med. 2014;370(20):1889-98.

18. Kowdley KV, Gordon SC, Reddy KR, Rossaro L, Bernstein DE, Lawitz E, et al. Ledipasvir and sofosbuvir for 8 or 12 weeks for chronic HCV without cirrhosis. N Engl J Med. 2014;370(20):1879-88.

19. Sulkowski MS, Gardiner DF, Rodriguez-Torres M, Reddy KR, Hassanein T, Jacobson I, et al. Daclatasvir plus sofosbuvir for previously treated or untreated chronic HCV infection. N Engl J Med. 2014;370(3):211-21.

20. Zeuzem S, Jacobson IM, Baykal T, Marinho RT, Poordad F, Bourliere M, et al. Retreatment of HCV with ABT-450/r-ombitasvir and dasabuvir with ribavirin. N Engl J Med. 2014;370(17):1604-14.

21. Lawitz E, Mangia A, Wyles D, Rodriguez-Torres M, Hassanein T, Gordon S, et al. Sofosbuvir for previously untreated chronic hepatitis C infection. N Engl J Med. 2013;368(20):1878-87.

22. Roy E, Haley N, Leclerc P, Sochanski B, Boudreau JF, Boivin JF. Mortality in a cohort of street youth in Montreal. JAMA. 2004;292(5):569-74.

23. Thein HH, Yi Q, Dore GJ, Krahn MD. Estimation of stage-specific fibrosis progression rates in chronic hepatitis C virus infection: a meta-analysis and meta-regression. Hepatology. 2008;48(2):418-31.

24. Salomon JA, Weinstein MC, Hammitt JK, Goldie SJ. Cost-effectiveness of treatment for chronic hepatitis C infection in an evolving patient population. JAMA. 2003;290(2):228-37.

25. Salomon JA, Weinstein MC, Hammitt JK, Goldie SJ. Empirically calibrated model of hepatitis C virus infection in the United States. Am J Epidemiol. 2002;156(8):761-73.

26. Singal AG, Volk ML, Jensen D, Di Bisceglie AM, Schoenfeld PS. A sustained viral response is associated with reduced liver-related morbidity and mortality in patients with hepatitis C virus. Clin Gastroenterol Hepatol. 2010;8(3):280-8, 8 e1.

27. Sutton AJ, Gay NJ, Edmunds WJ, Hope VD, Gill ON, Hickman M. Modelling the force of infection for hepatitis B and hepatitis C in injecting drug users in England and Wales. BMC Infect Dis. 2006;6:93.

28. Martin NK, Hickman M, Miners A, Hutchinson SJ, Taylor A, Vickerman P. Cost-effectiveness of HCV case-finding for people who inject drugs via dried blood spot testing in specialist addiction services and prisons. BMJ Open. 2013;3(8).

29. Mathers BM, Degenhardt L, Bucello C, Lemon J, Wiessing L, Hickman M. Mortality among people who inject drugs: a systematic review and meta-analysis. Bull World Health Organ. 2013;91(2):102-23.

30. Miller CL, Kerr T, Strathdee SA, Li K, Wood E. Factors associated with premature mortality among young injection drug users in Vancouver. Harm Reduct J. 2007;4:1.

31. Tran VC. Une ballade en forêts aléatoires: Université Lille 1; 2014.

32. Andersson H, Britton T. Stochastic epidemic models and their statistical analysis: Springer Science & Business Media; 2012.
